# Supplementary material for: A Network of MicroRNAs and mRNAs Involved in Melanosome Maturation and Trafficking Defines the Lower Response of Pigmentable Melanoma Cells to Targeted Therapy
Source: Cancers (Basel). 2023 Jan 31;15(3):894. doi: 10.3390/cancers15030894 (PMC9913661; doi:10.3390/cancers15030894)
Supplement: Supplementary file 1 [file cancers-15-00894-s001.zip › Supplementary Material 311022.pdf]

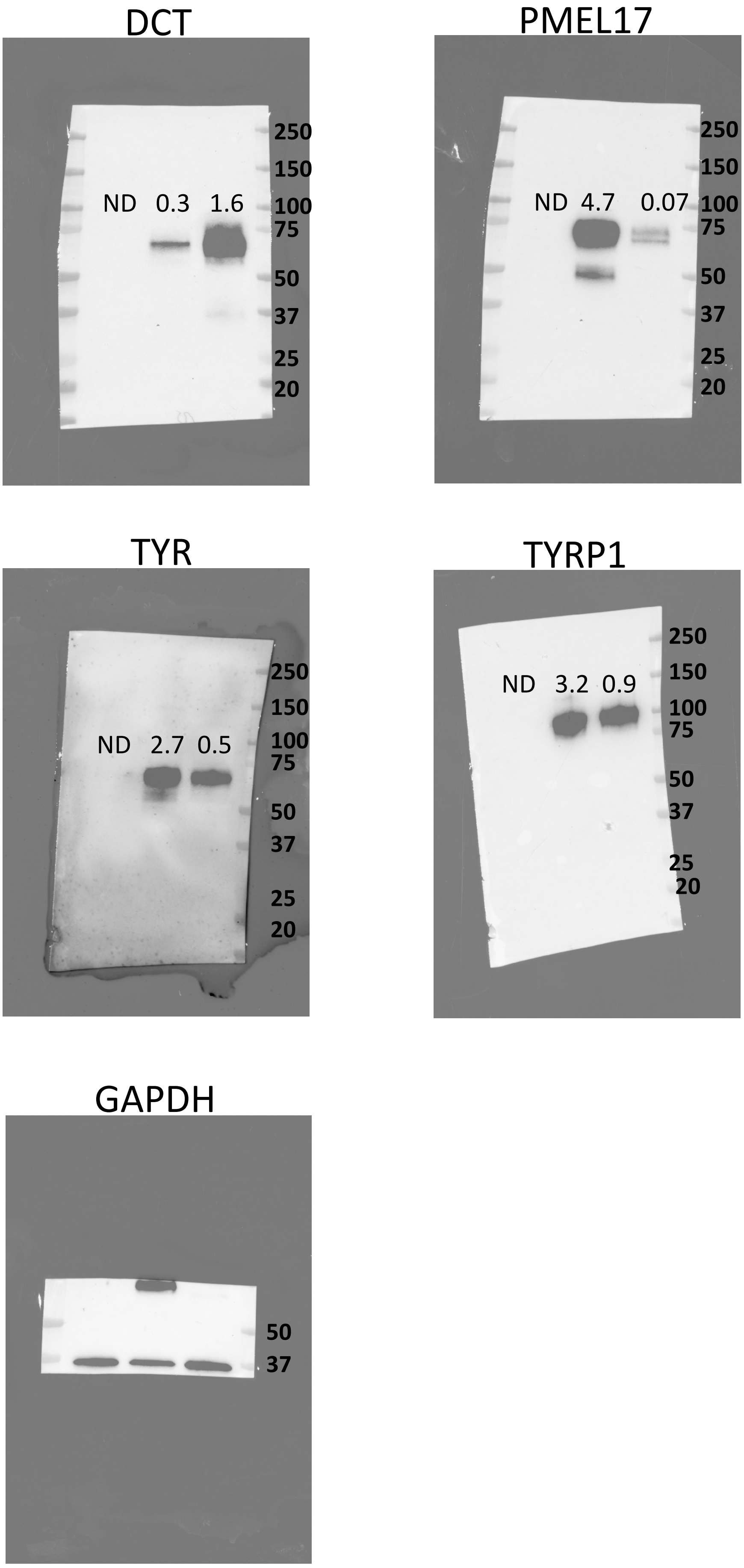

Supplementary Material. Whole blots and densitometry readings of the western blot presented in Fig.1c.
The whole blot and the densitometry readings are reported for each protein detected. Marker size is reported on the right. ND: not detected.
