# Supplementary material for: A Network of MicroRNAs and mRNAs Involved in Melanosome Maturation and Trafficking Defines the Lower Response of Pigmentable Melanoma Cells to Targeted Therapy
Source: Cancers (Basel). 2023 Jan 31;15(3):894. doi: 10.3390/cancers15030894 (PMC9913661; doi:10.3390/cancers15030894)
Supplement: Supplementary file 1 [file cancers-15-00894-s001.zip › Supplementary-figures.pdf]

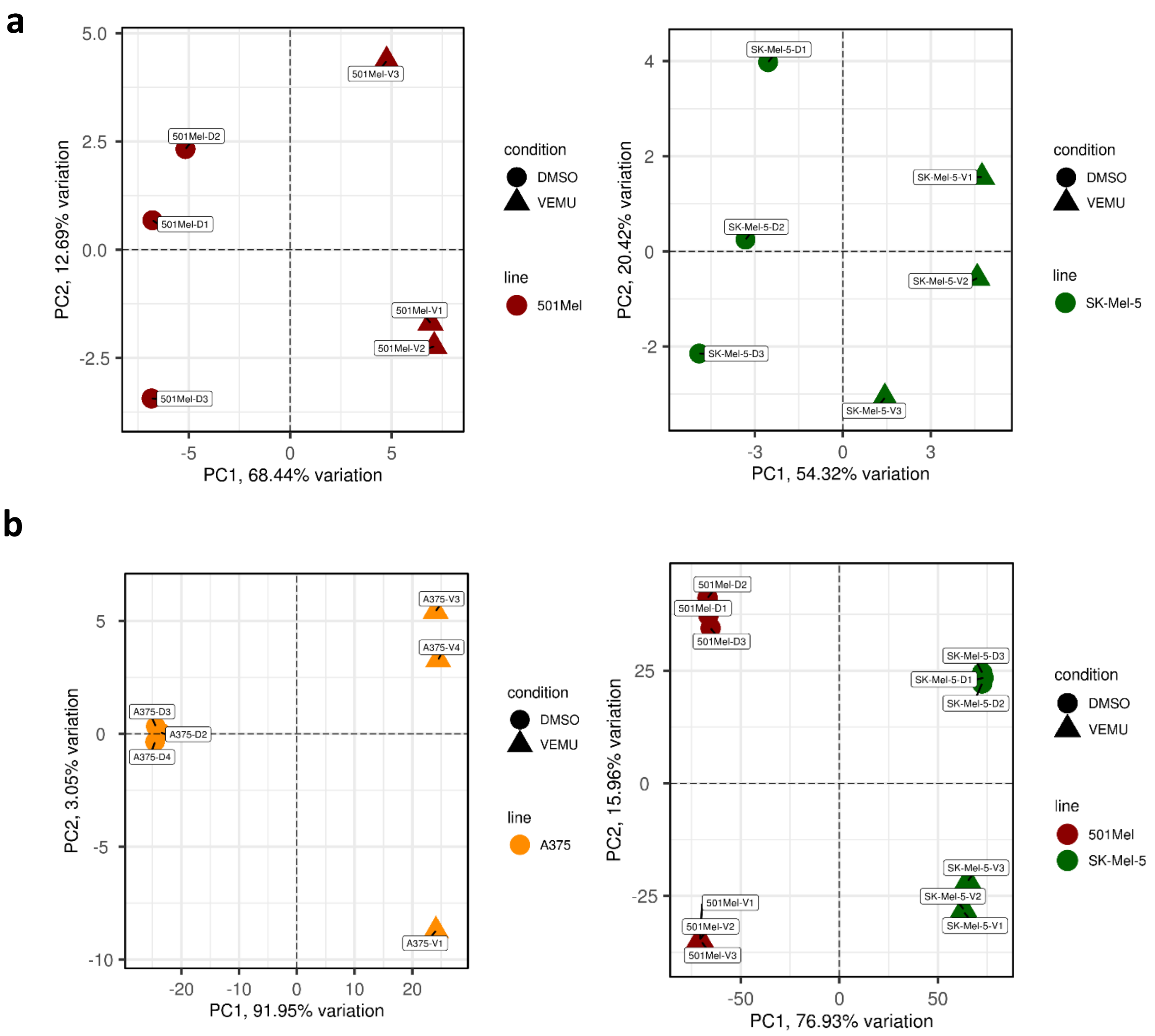


**Supplementary Figure S1. PCA analysis.**
Principal component analysis (PCA) shows low variance among biological replicates (#1-3), attesting the reproducibility of drug treatment protocol.
**a.** smallRNA-seq of 501Mel (*left*), and SK-Mel-5 (*right*).
**b.** RNA-seq of A375 cells (GSE89129, *left*), and RNA-seq of 501Mel and SK-Mel-5 cells (*right*).
D: DMSO; V: vem.

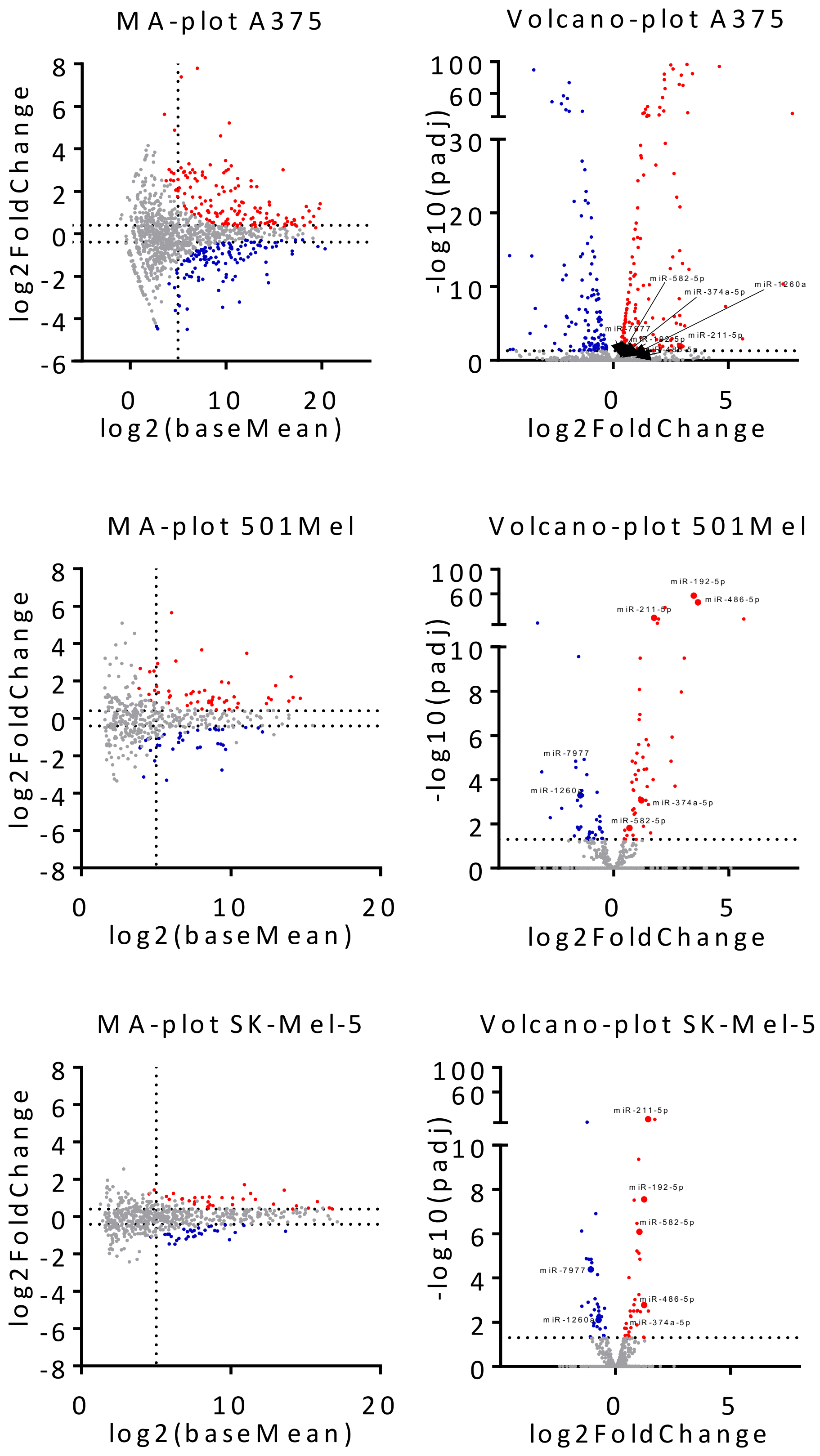


**Supplementary Figure S2. smallRNA-seq analysis.**

Logarithmic scatter plots (MA-plots, *left*), showing miRNA FoldChange (log2FoldChange, *y axis*) as a function of average number of reads (log2(baseMean), *x axis*), and Volcano plots (*right*), showing the p-value of differentially expressed miRNAs (-log10(padj), *y axis*) as a function of fold change (log2(FoldChange), *x axis*). A375 (*top*), 501Mel (*middle*) and SK-Mel-5 (*bottom*) are shown.

In MA-plots, the cutoff on average number of reads is 32 (log2(baseMean)=5), with average number of reads defined as mean of read number in all 6 samples (3 DMSO and 3 vem replicates). The cutoff on FoldChange is 0.4 (log2FoldChange>=0.4 or log2FoldChange<=-0.4. In Volcano plots, the cutoff on adjusted p-value is 0.05 (-log10(padj)=1.3). According to all these cutoffs, the DE miRNAs are those in red (up-regulated in vem compared to DMSO) and blue (down-regulated in vem compared to DMSO).

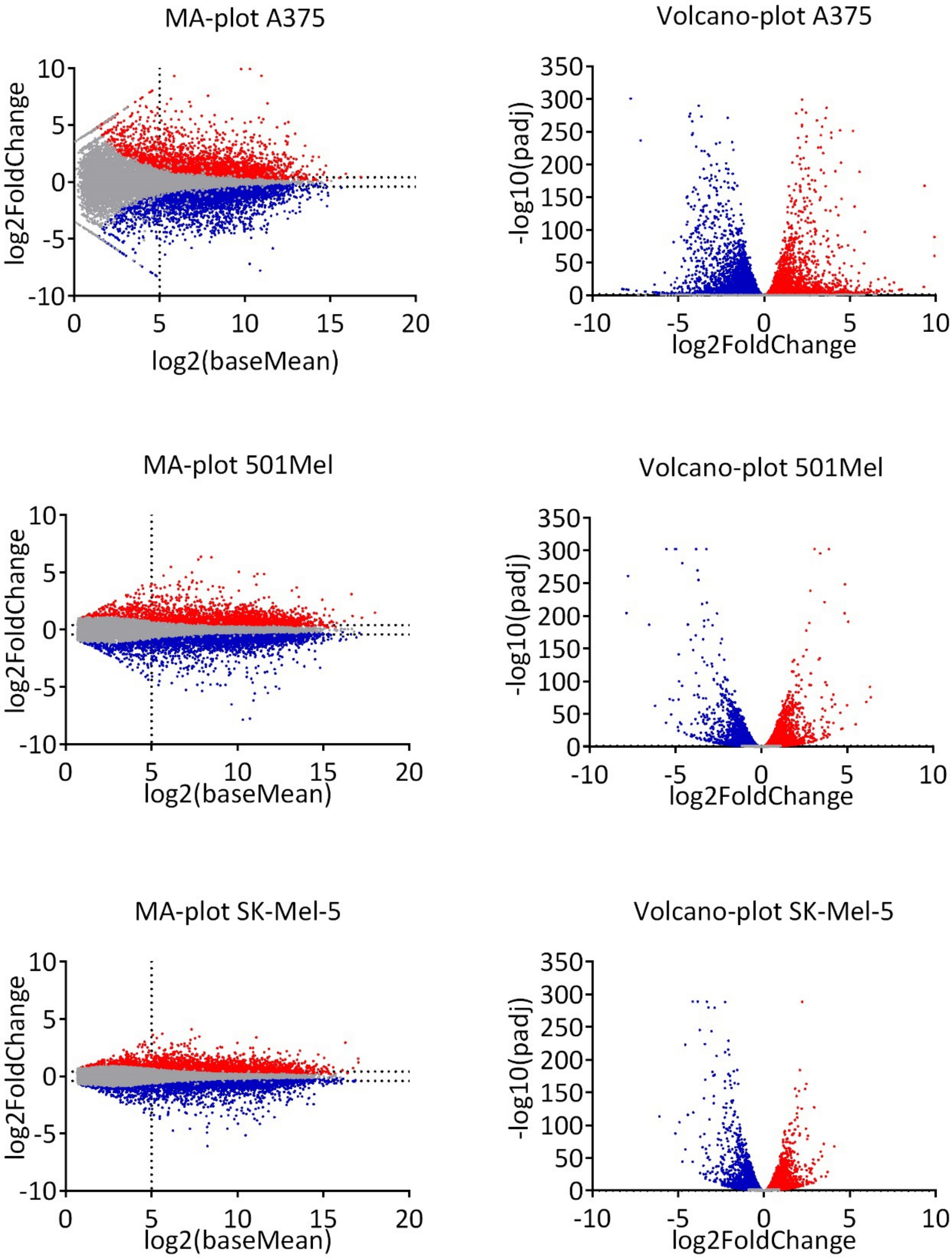


**Supplementary Figure S3. RNA-seq analysis.**

Logarithmic scatter plots (MA-plots, *left*), showing RNA FoldChange (log2FoldChange, *y axis*) as a function of average number of reads (log2(baseMean), *x axis*), and Volcano plots (*right*), showing the p-value of differentially expressed RNAs (-log10(padj), *y axis*) as a function of fold change (log2(FoldChange), *x axis*). A375 (*top*), 501Mel (*middle*) and SK-Mel-5 (*bottom*) are shown.

In MA-plots, the cutoff on average number of reads is 32 (log2(baseMean)=5), with average number of reads defined as mean of read number in all 6 samples (3 DMSO and 3 vem replicates). The cutoff on FoldChange is 0.4 (log2FoldChange>=0.4 or log2FoldChange<=-0.4. In Volcano plots, the cutoff on adjusted p-value is 0.05 (-log10(padj)=1.3). According to all these cutoffs, the DE RNAs are those in red (up-regulated in vem compared to DMSO) and blue (down-regulated in vem compared to DMSO).

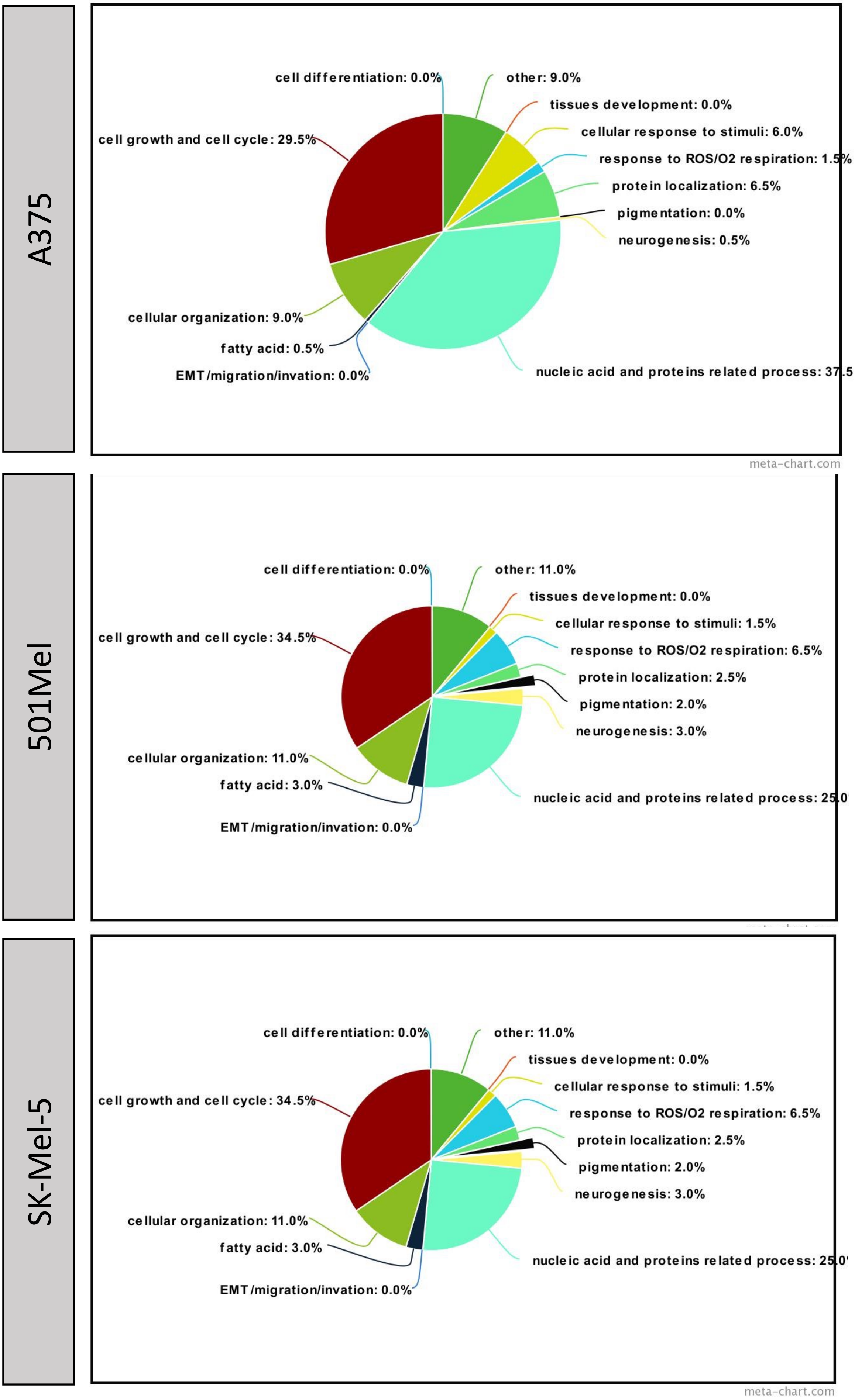


**Supplementary Figure S4. Pie chart of the 12 macroBPs in A375 (top), 501Mel (middle) and SK-Mel-5 (bottom) cells.**

**a**

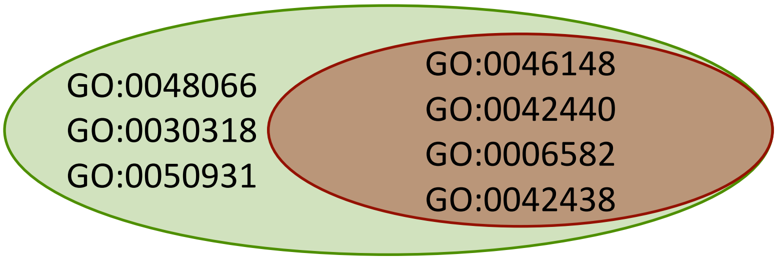


**b**

| termID | termName | termDefinition | p.adjusted | geneSymbol |
|---|---|---|---|---|
| GO:0046148 | pigment biosynthetic process | The chemical reactions and pathways resulting in the formation of a pigment, any general or particular coloring matter in living organisms, e.g. melanin. | 0.00032072 | *DCT OCA2 TYRP1 UROS TRPC1 PMEL CTNS SLC7A11 ALAS1 FECH DDT ZEB2 CDH3 SLC45A2 IBA57 COX10 CITED1 GPR143 SLC25A39 GIPC1 ALAD TYR IREB2 COX15 SLC24A5 PGRMC1 SHMT1 GMPS APRT PPAT ADA CHML CHML PAICS PRPS1 MC1R CPOX HPRT1* |
| GO:0042440 | pigment metabolic process | The chemical reactions and pathways involving pigment, any general or particular coloring matter in living organisms, e.g. melanin. | 0.00081532 | *DCT OCA2 TYRP1 PPARGC1A UROS TRPC1 BDH2 PMEL CTNS SLC7A11 ALAS1 FECH DDT ZEB2 BLVRB CDH3 SLC45A2 IBA57 COX10 CITED1 HMOX2 GPR143 ABCC2 SLC25A39 GIPC1 HMOX1 ALAD BCL2 TYR IREB2 COX15 SLC24A5 PGRMC1 SHMT1 GMPS APRT PPAT ADA CHML CHML PAICS PRPS1 MC1R CPOX HPRT1* |
| GO:0048066 | developmental pigmentation | The developmental process that results in the deposition of coloring matter in an organism, tissue or cell. | 0.00089468 | *TYRP1 KIT DCT OCA2 CITED1 RAB27A SLC45A2 GPR143 RAB38 EDNRB RAB32 ZEB2 MITF TYR BLOC1S3 GLI3 MREG BCL2 KITLG MEF2C ENPP1* |
| GO:0006582 | melanin metabolic process | The chemical reactions and pathways involving melanins, pigments largely of animal origin. High molecular weight polymers of indole quinone, they are irregular polymeric structures and are divided into three groups: allomelanins in the plant kingdom and eumelanins and phaeomelanins in the animal kingdom. | 0.00115754 | *DCT OCA2 TYRP1 TRPC1 PMEL CTNS SLC7A11 DDT ZEB2 CDH3 SLC45A2 CITED1 GIPC1 BCL2 TYR SLC24A5 CHML CHML MC1R* |
| GO:0042438 | melanin biosynthetic process | The chemical reactions and pathways resulting in the formation of melanins, pigments largely of animal origin. High molecular weight polymers of indole quinone, they are irregular polymeric structures and are divided into three groups: allomelanins in the plant kingdom and eumelanins and phaeomelanins in the animal kingdom. | 0.00263844 | *DCT OCA2 TYRP1 TRPC1 PMEL CTNS SLC7A11 DDT ZEB2 CDH3 SLC45A2 CITED1 GIPC1 TYR SLC24A5 CHML CHML MC1R* |
| GO:0030318 | melanocyte differentiation | The process in which a relatively unspecialized cell acquires specialized features of a melanocyte. | 0.0018284 | *TYRP1 KIT OCA2 CITED1 RAB27A EDNRB ZEB2 MITF GLI3 MREG BCL2 KITLG MEF2C ENPP1* |
| GO:0050931 | pigment cell differentiation | The process in which a relatively unspecialized cell acquires the specialized features of a pigmented cell, such as a melanocyte. | 0.00191497 | *TYRP1 KIT OCA2 CITED1 RAB27A RAB38 EDNRB RAB32 ZEB2 MITF BLOC1S3 GLI3 MREG BCL2 KITLG MEF2C ENPP1* |

**Supplementary Figure S5. The 7 BPs belonging to the macroBP «pigmentation» and relative genes.**

**a.** BPs belonging to the macroBP «pigmentation» in 501Mel and SK-Mel-5 cells. The 4 BPs of 501Mel cells (dark red) are a subgroup of the 7 BPs of SK-Mel-5 cells (olive green).

**b.** The 7 BPs encompass a total of 64 genes, 15 of which are DEmRNAs (underlined).

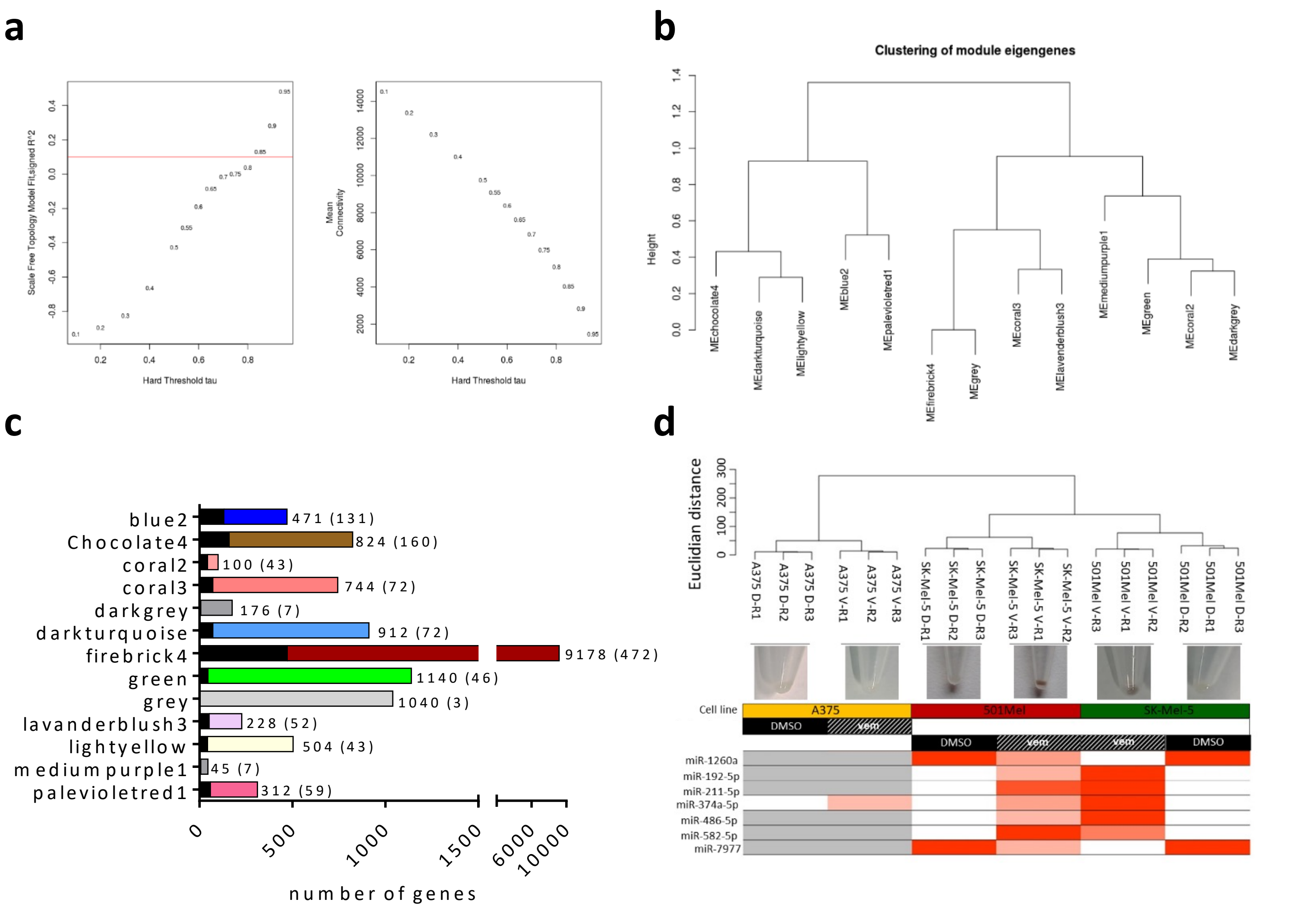


**e**

| module | termID | termName | termDefinition | pvalue | geneSymbol |
|---|---|---|---|---|---|
| chocolate4 | GO:0048066 | developmental pigmentation | The developmental process that results in the deposition of coloring matter in an organism, tissue or cell. | 0.00825 | *BAX DCT ENPP1 OCA2 RAB27A RAB32 TYRP1* |
| | GO:0006582 | melanin metabolic process | The chemical reactions and pathways involving melanins, pigments largely of animal origin. High molecular weight polymers of indole quinone, they are irregular polymeric structures and are divided into three groups: allomelanins in the plant kingdom and eumelanins and phaeomelanins in the animal kingdom. | 0.03719 | *DCT OCA2 OPN3 TYRP1* |
| | GO:0030318 | melanocyte differentiation | The process in which a relatively unspecialized cell acquires specialized features of a melanocyte. | 0.03719 | *ENPP1 OCA2 RAB27A TYRP1* |
| | GO:0042438 | melanin biosynthetic process | The chemical reactions and pathways resulting in the formation of melanins, pigments largely of animal origin. High molecular weight polymers of indole quinone, they are irregular polymeric structures and are divided into three groups: allomelanins in the plant kingdom and eumelanins and phaeomelanins in the animal kingdom. | 0.03210 | *DCT OCA2 OPN3 TYRP1* |

**Supplementary Figure S6. WGCNA analysis.**

**a.** Analysis of scale free topology for multiple hard thresholds to select the appropriate threshold for network construction. The chosen value is 0.85.

**b.** Hierarchical clustering dendrogram of module eigengenes (labeled by their color).

**c.** Total number of genes contained in each module. DEmRNAs present in each module are highlighted in black within the bars, while their number is reported in brackets.

**d.** Dendrogram (*upper*) and traits (treatment (DMSO and vem, *middle*); DEmiR expression (*bottom*)). For each sample type, a representative picture of cell pellet is shown.

**e**. Pigmentation-related GO:BPs belonging to the *chocolate4* module. The 4 BPs encompass a total of 8 genes, 4 of which are DEmRNAs (underlined).

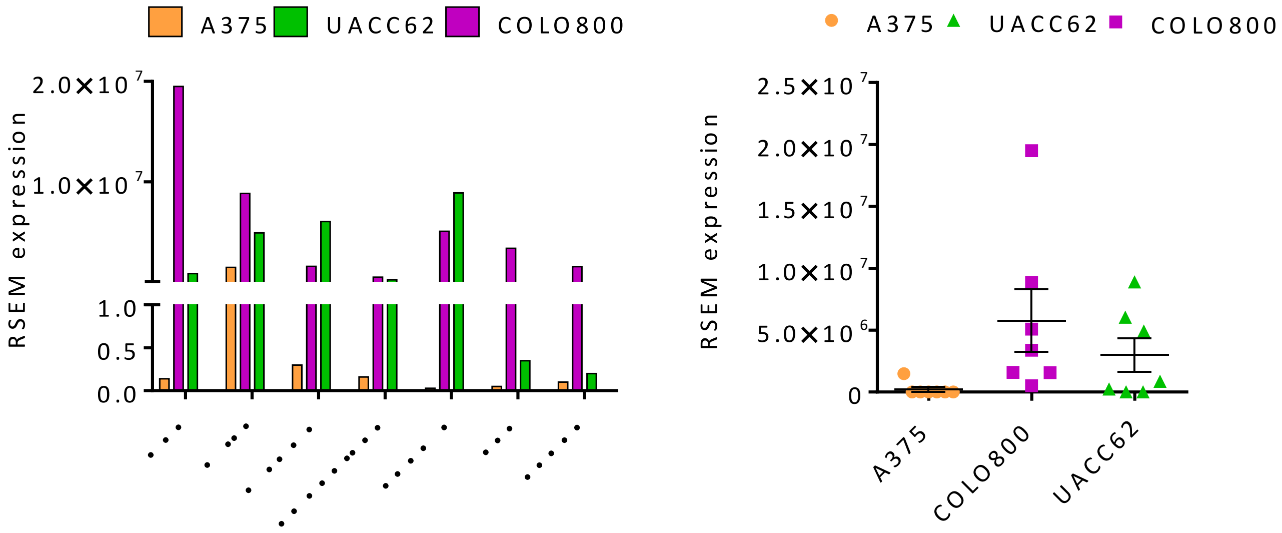


**Supplementary Figure S7. mRNA expression level of genes involved melanin synthesis, as detected in pigmentable UACC62 and Colo800 melanoma cells.**

mRNA level (*left*, single genes; *right*, grouped genes) of genes involved melanin synthesis, as detected in pigmentable melanoma cells (UACC62 (green) and COLO800 (purple)) and in non-pigmentable A375 melanoma cells (orange). CCLE data (https://sites.broadinstitute.org/ccle/) are reported.

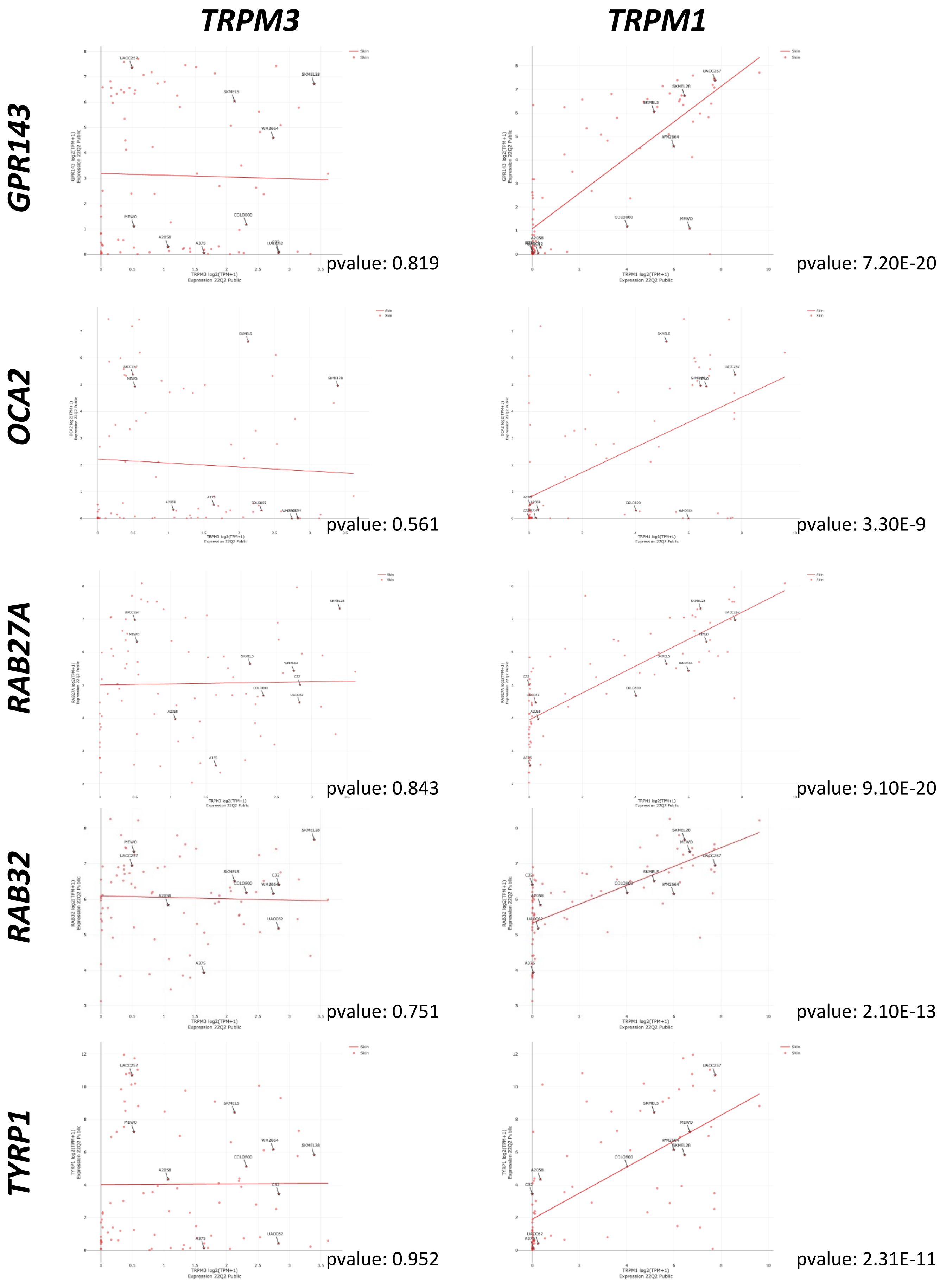


**Supplementary Figure S8. Correlation between the expression level of the indicated mRNAs in the melanoma cell lines present at CCLE.**
The analysis was performed using the data explorer tool of the Dependency Map web portal (see M&M section).

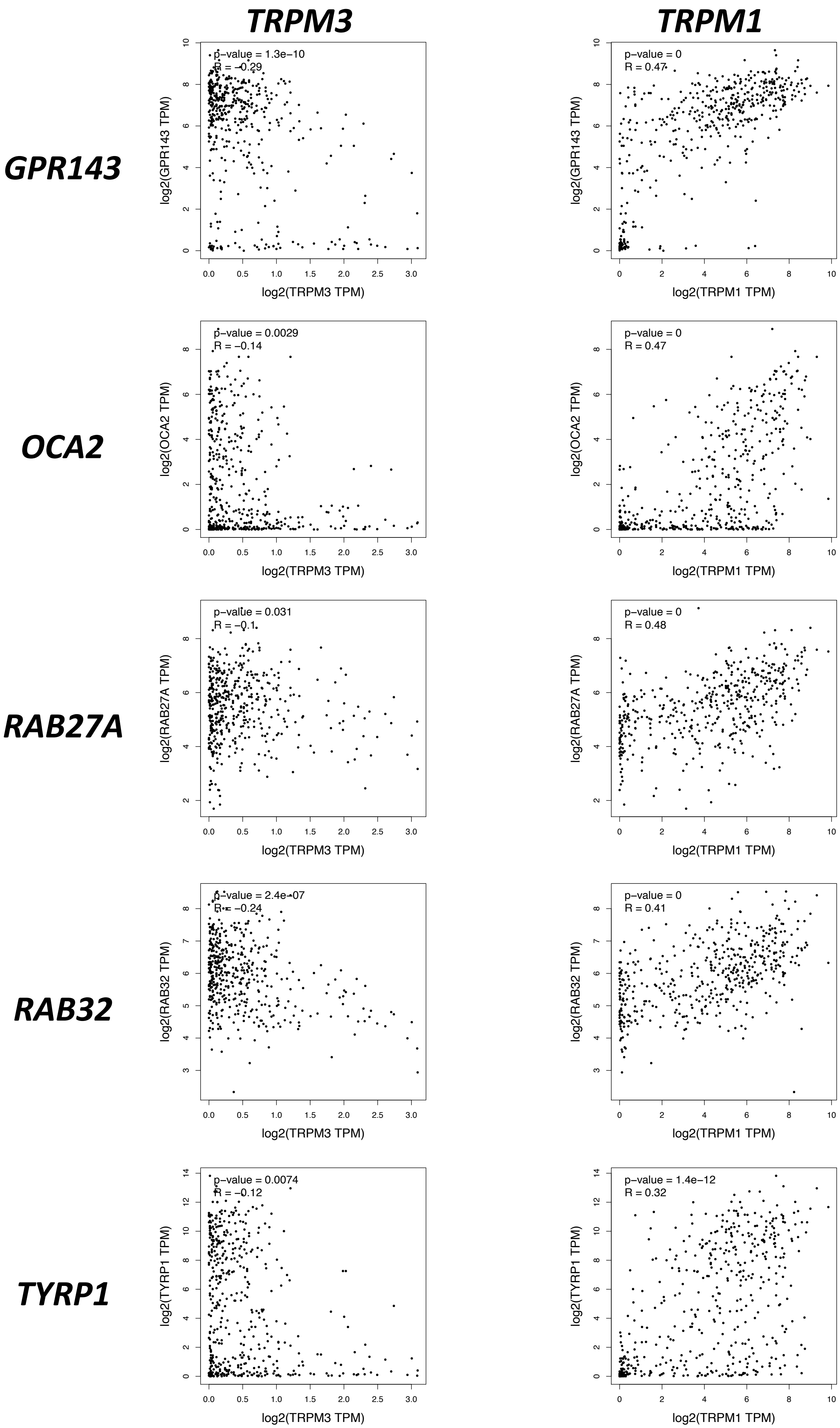


**Supplementary Figure S9. Correlation between the expression level of the indicated mRNAs in the SKCM dataset.** The analysis was performed using GEPIA (http://gepia.cancer-pku.cn/).

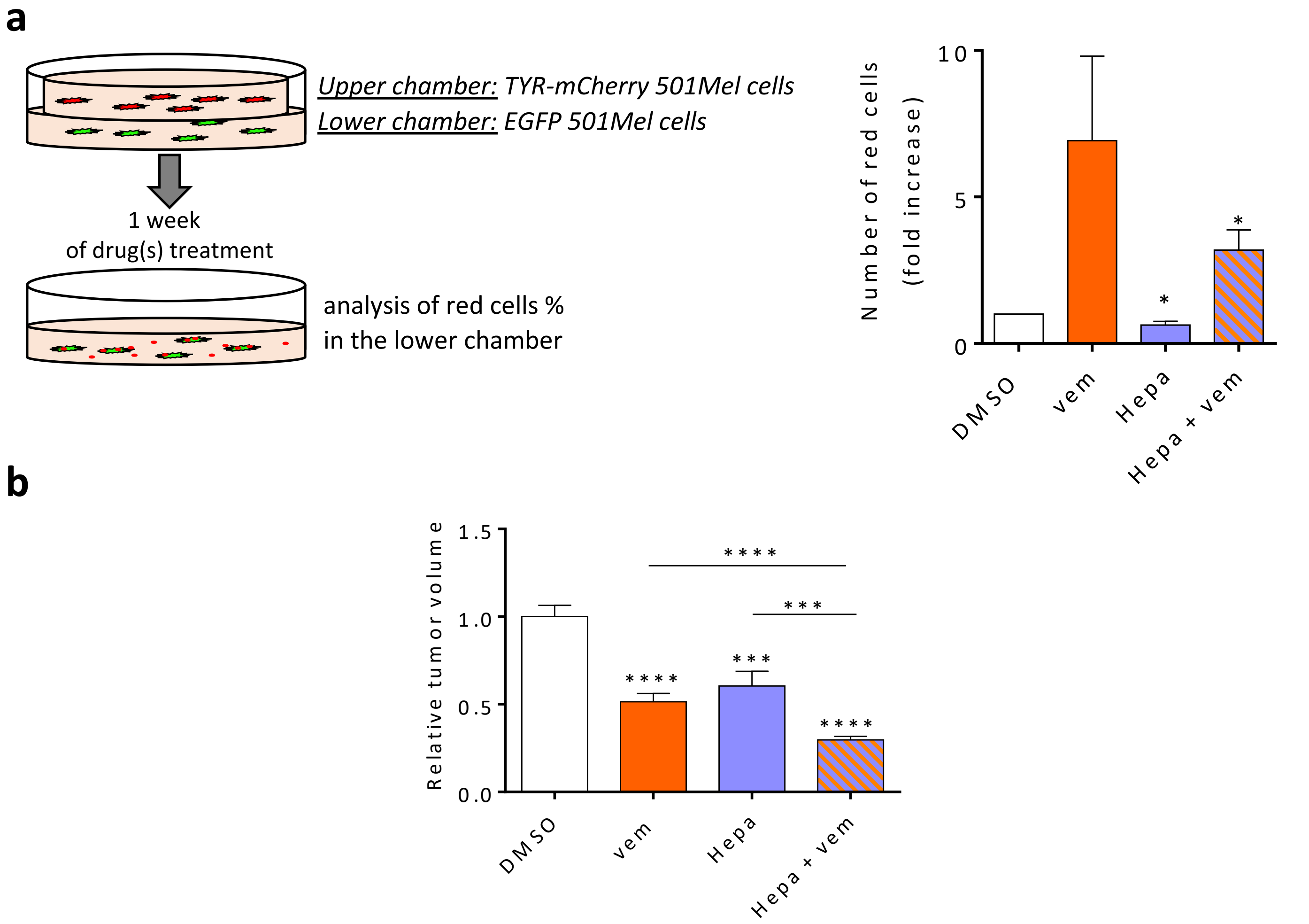


**Supplementary Figure S10. Melanosome uptake and sensitivity to vem of 501Mel cells treated with Heparin (Hepa).**

**a.** Melanosome uptake. (*left*) TYR-mCherry 501Mel cells (*upper chamber*) were co-cultured with EGFP 501Mel cells (*lower chamber*) and treated with the indicated drugs (5uM vem, 10ug/ml Heparin (Hepa), 5uM vem + 10ug/ml Hepa), or left untreated (DMSO vehicle). (*right*). The % of red cells present in the lower chamber was measured by flow cytometry after one week.

**b.** Sensitivity to vem. TYR-mCherry 501Mel cells were treated for 48h with the indicated drugs (0.2uM vem, 10ug/ml Hepa, 0.2uM vem + 10ug/ml Hepa), or left untreated (DMSO vehicle). They were then injected into the yolk sac of 48hpf zebrafish embryos. The volume of the xenografted tumors was measured 48h later.

Graphs represent the mean±SEM of 3 independent experiments. *p<0.05, ***p<0.001, ****p<0.0001.

a

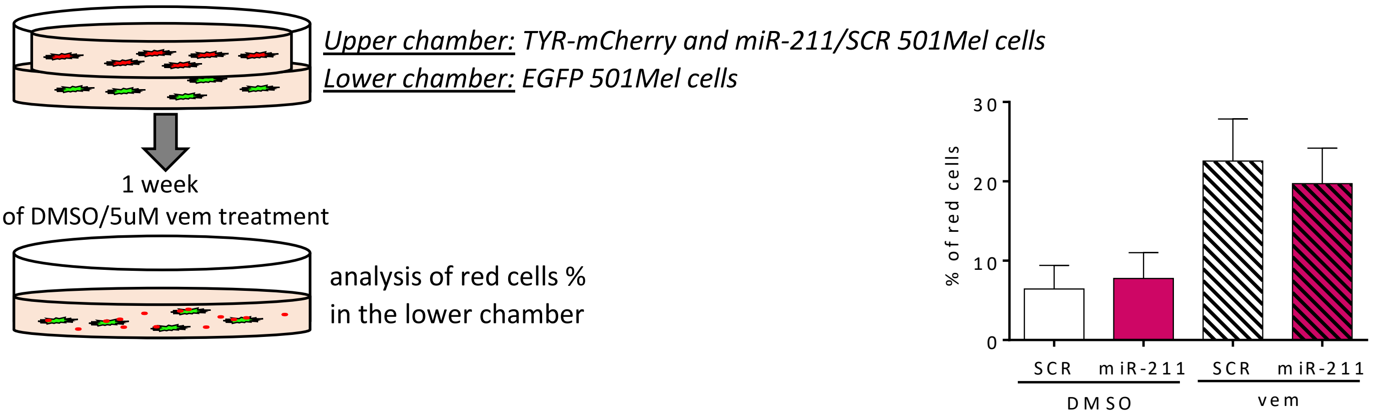


b

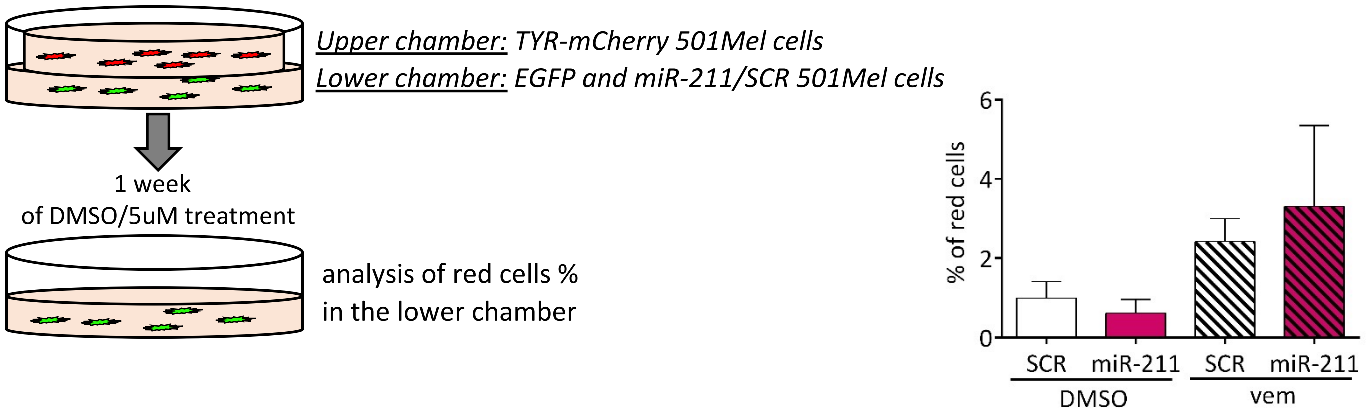


**Supplementary Figure S11. Melanosome release and uptake in 501Mel cells overexpressing miR-211.**

**a.** Melanosome release. (*left*) TYR-mCherry and miR-211/SCR over-expressing 501Mel cells (*upper chamber*) were co-cultured with EGFP 501Mel cells (*lower chamber*), and treated with 5uM vem or DMSO vehicle. (*right*) The % of red cells present in the lower chamber was measured by flow cytometry after one week.

**b.** Melanosome uptake. (*left*) TYR-mCherry 501Mel cells (*upper chamber*) were co-cultured with EGFP and miR-211/SCR over-expressing 501Mel cells (*lower chamber*), and treated with 5uM vem or DMSO vehicle. (*right*) The % of red cells present in the lower chamber was measured by flow cytometry after one week.

Graphs represent the mean±SEM of 3 independent experiments.
